# Evaluation of the concomitant use of prophylactic treatments in patients with migraine under anti‐calcitonin gene‐related peptide therapies: The PREVENAC study

**DOI:** 10.1111/ene.16215

**Published:** 2024-02-07

**Authors:** Ana Beatriz Gago‐Veiga, Noelia Lopez‐Alcaide, Sonia Quintas, Iris Fernández Lázaro, Javier Casas‐Limón, Carlos Calle, Germán Latorre, Nuria González‐García, Jesús Porta‐Etessam, Jaime Rodriguez‐Vico, Alex Jaimes, Andrea Gómez García, David García‐Azorín, Ángel Luis Guerrero‐Peral, Álvaro Sierra, Alberto Lozano Ros, Antonio Sánchez‐Soblechero, Javier Díaz‐de‐Teran, Javier A. Membrilla, Cristina Treviño, Alicia Gonzalez‐Martinez

**Affiliations:** ^1^ Headache Unit Hospital Universitario de la Princesa & Instituto de Investigación Sanitaria Princesa (IIS‐Princesa) Madrid Spain; ^2^ Headache Unit Hospital Universitario Fundación Alcorcón Alcorcón Spain; ^3^ Headache Unit Hospital de Fuenlabrada Madrid Spain; ^4^ Headache Unit Hospital Clínico San Carlos Madrid Spain; ^5^ Headache Unit Hospital Fundación Jiménez Díaz Madrid Spain; ^6^ Headache Unit Hospital Clínico Universitario de Valladolid Valladolid Spain; ^7^ Department of Medicine Universidad de Valladolid Valladolid Spain; ^8^ Headache Unit Hospital Universitario Gregorio Marañón Madrid Spain; ^9^ Headache Unit Hospital Severo Ochoa Madrid Spain; ^10^ Headache Unit Hospital Clínico Universitario de la Paz Madrid Spain

**Keywords:** CGRP, effectiveness, migraine, oral prophylactics, response

## Abstract

**Background and purpose:**

Anti‐calcitonin gene‐related peptide (CGRP) therapies are recent preventive therapies approved for both episodic and chronic migraine. One of the measures of effectiveness is the withdrawal of other preventive treatments. The objective of this study is to quantify the impact of anti‐CGRP drugs in concomitant preventive treatment in patients with migraine.

**Methods:**

This was an observational, retrospective, multicenter cohort study with patients from nine national headache units. Patients with migraine undergoing treatment for at least 6 months with anti‐CGRP antibodies, who were initially associated with some preventive treatment (oral and/or onabotulinumtoxinA) were included. Demographic and clinical variables were collected, as well as variables related to headache. Differences according to withdrawal or nonwithdrawal were evaluated.

**Results:**

A total of 408 patients were included, 86.52% women, 48.79 (SD = 1.46) years old. Preventive treatment was withdrawn in 43.87% (179/408), 20.83% partially and 23.04% totally. In 27.45% (112/408), it was maintained exclusively due to comorbidity and in 28.6% (117/408) due to partial efficacy. The most frequent time of withdrawal was between 3 and 5 months after the start of treatment. The baseline characteristics associated with nonwithdrawal were comorbidities: insomnia, hypertension and obesity, chronic migraine, and medication overuse. In the multivariate analysis, the absence of high blood pressure, a greater number of preventive treatments at the start, and a lower number of migraine days/month after anti‐CGRP treatment were independently associated with withdrawal of the treatment (*p* < 0.05).

**Conclusions:**

Anti‐CGRP antibodies allow the withdrawal of associated preventive treatment in a significant percentage of patients, which supports its effectiveness in real‐life conditions.

## INTRODUCTION

Migraine is a disabling disease affecting more than 1 billion people worldwide [1, 2]. Moreover, it is one of the leading causes of disability in young adults [2, 3]. Among patients with migraine, approximately 1%–2% suffer chronic migraine (CM), which produces a pronounced decline in both quality of life and functional capacity [4, 5].

Preventive medication therapy is generally used to reduce the frequency, duration, or severity of attacks [6], ultimately enhancing overall quality of life. However, according to recent observational studies, only 25% of patients are on preventive therapies despite being subject of beneficial effect if treated [7, 8]. Furthermore, many conventional oral prophylactic treatments exhibit poor adherence and a considerable incidence of adverse events, restricting their usage [9].

In the past decade, effective and specific migraine prophylactic treatments have emerged for both episodic migraine (EM) and CM with the potential of reducing the global burden of disease [10]. Currently, there are four anti‐calcitonin gene‐related peptide (CGRP) monoclonal antibodies available (erenumab, galcanezumab, fremanezumab, and eptinezumab) that have demonstrated effectiveness and safety in both clinical trials and real‐world studies [11, 12, 13] and are currently used in clinical practice [14]. Earlier research has shown that successful preventive medications, such as onabotulinumtoxinA (OnabotA), substantially curtail the need for concurrent oral treatments, underscoring their effectiveness [15]. However, up until now, no studies have explored the impact of recent anti‐CGRP therapies on concurrent preventive regimens.

The present study aims to quantify changes in the utilization of concurrent prophylactic medications following the initiation of anti‐CGRP therapy among patients with EM and CM.

## METHODS

### Study design and patient selection

We conducted a retrospective, multicentric, cross‐sectional study conducted in nine headache units across Spain including patients with EM and CM from February 2020 to February 2023. Patients aged >18 years with a history of EM or CM (≥15 headache days per month, of which ≥8 were migraine days; with or without aura) according to the International Classification of Headache Disorders criteria [16], taking preventive treatments at the time of anti‐CGRP treatment start, with a minimum duration of anti‐CGRP therapies of 6 months, and with <20% of data missing were included in the study. Patients with a diagnosis of medication overuse (MO) were permitted to participate in the study. Anti‐CGRP therapies that are indicated for preventive treatment of EM or CM in adults and approved in multiple jurisdictions (Europe [European Medicines Agency and the United States [US Food and Drug Administration]) were prescribed according to the criteria of the neurologist in charge. Anti‐CGRP receptor and ligand antibody treatments were approved in Europe with the associated costs being covered in Spain by the Spanish National Health System, for patients with 8 or more migraine days/month (high‐frequency EM and CM) for whom treatment with three preventive medications at sufficient doses for at least 3 months had failed, one of which being OnabotA in the case of CM [17, 18, 19]. In clinical practice, withdrawal of other preventive treatments before anti‐CGRP receptor or ligand antibody treatment start is not mandatory, and therefore it may be used as an “add‐on” therapy at onset.

### Study procedures

Clinical and demographic variables including sex, age, age at onset, migraine diagnosis (EM, CM), type of migraine (with or without aura), migraine duration (years), CM duration (months), MO, anti‐CGRP drug used (erenumab, galcanezumab, fremanezumab), number of prior preventive treatments (OnabotA and oral preventive treatments), number of concomitant preventive treatments at anti‐CGRP start, psychiatric comorbidities (anxiety or depression), withdrawal of preventive treatments, and main reason for continuation such as comorbidities (insomnia, hypertension, anxiety/depression, obesity) or discontinuation such as effectiveness of anti‐CGRP monoclonal antibodies were collected from the patients' medical history.

Headache characteristics were thoroughly assessed in the study. This encompassed scores obtained from the Headache Impact Test (HIT‐6 scale) [20], a straightforward and widely utilized tool in clinical settings for quantifying disability linked to headache [21, 22, 23], the psychiatric comorbidities anxiety and depression, baseline number of headache days per month, and baseline number of migraine days per month were collected at baseline and at the time of inclusion in the study. Additionally, the 30%, 50%, and 75% response rate regarding monthly headache days (MHD; number of days with headache per month) and monthly migraine days (MMD; number of days with headache with migraine characteristics with a duration of ≥4 h OR triptan intake [independently from its effect]) was also calculated.

The primary endpoint of the current study centered on determining the percentage of patients who discontinued any form of preventive treatments. Based on this criterion, individuals were categorized into two groups: patients with complete withdrawal of all their initial preventive treatments and those with partial withdrawal considered as a reduction in the treatment dosage or a withdrawal of one of the preventive therapies in case there are many, after commencing anti‐CGRP therapies. Furthermore, the study delved into secondary endpoints, including the timing of concurrent treatment discontinuation, factors linked to withdrawal, and the rationale behind maintaining certain treatments.

### Statistical analysis

The descriptive analyses and group comparisons were carried out using IBM SPSS Statistics v21.0 software. For the descriptive analyses of the clinical and treatment outcome variables, counts (*n*) and percentages were used as measures of frequency for categorical or nominal variables. We checked the normality assumption of quantitative variables using the Shapiro–Wilk test. Quantitative variables are summarized as mean and SD as a measure of central tendency and dispersion, respectively (mean ± SD), and qualitative variables as frequencies and percentages.

To evaluate the effectiveness of anti‐CGRP treatment and to determine differences in outcome measures between patients with and without withdrawal of preventive treatments, we analyzed data using a Pearson chi‐squared or Fisher exact test for categorical variables, the linear trend chi‐squared test for ordinal variables, and the Student *t*‐test or Mann–Whitney *U*‐test for quantitative variables. Paired *t*‐tests were used to measure statistical differences in patient headache frequency before and after anti‐CGRP treatment. Independent predictors associated with prophylactic treatment withdrawal were also assessed using a logistic regression multivariate model including variables that were statistically significant (*p* < 0.1) in the univariate analysis. The analysis involved two‐tailed tests with presented *p*‐values, considering those <0.05 as statistically significant. A statistical power calculation was not performed prior to the study due to sample size being based on available data.

The institutional research ethics committee of the Hospital Universitario de La Princesa approved the research protocol of this study, and the study was conducted in accordance with the ethical principles of the Declaration of Helsinki.

## RESULTS

### Participants

**FIGURE 1:**
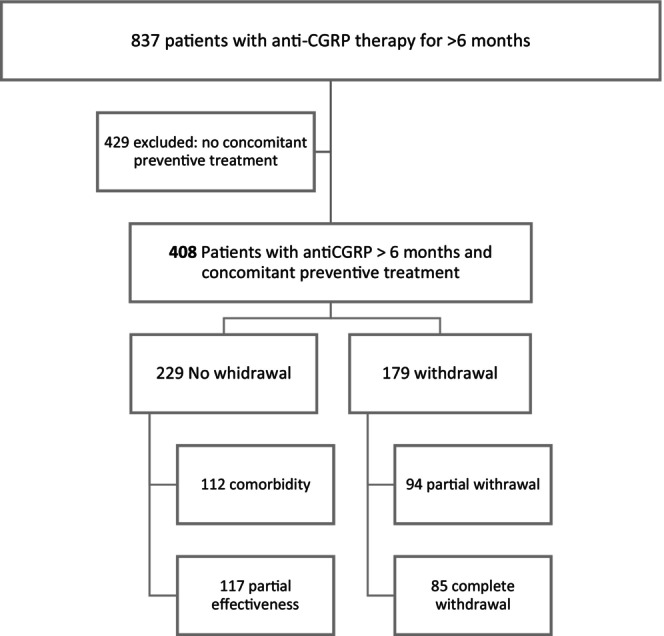
Flow diagram of the selection of patients and groups with respect to the withdrawal or nonwithdrawal of preventive treatment.

Among a total of 837 patients who were reviewed from 2020 to February 2023, 408 were included in our study according to the inclusion criteria (Figure 1). Only patients who were receiving concomitant preventive treatment were considered in the analysis.

Among them, 353 of 408 (86.52%) were female, with a mean age of 48.79 ± 11.46 years. Average time between diagnosis of CM and the start of anti‐CGRP therapy was 121.39 ± 93.06 months. Of 837 patients, 408 (48.75%) were receiving concomitant preventive treatment when anti‐CGRP was initiated, mean time with anti‐CGRP therapies was 16.07 ± 8.57 months, prior number of prophylactic treatments was 8.64 ± 3.72, and mean number of prophylactic treatments was 1.60 ± 0.91. Among patients under coexisting preventive therapies 230 of 408 (56%) were on one, 123 of 408 (30%) were on two, 39 of 408 (10%) were on three, and 16 of 408 (4%) were on four or more. Regarding headache variables, mean MMD at baseline was 15.02 ± 7.01 and mean MHD at baseline was 22.25 ± 7.58. The main demographic and baseline data are shown in Table 1.

**TABLE 1:** Baseline characteristics and headache variables in patients with anti‐CGRP drugs according to the presence of concomitant preventive treatment.

Baseline characteristics	Partial or total withdrawal	No withdrawal	*p*
Age, years, mean (SD)	47.56 (11.13)	49.76 (11.63)	0.054
Sex			
Women, *n*/*N* (%)	156/179 (87.15%)	197/229 (86.03%)	0.741
Men, *n*/*N* (%)	23/179 (12.85%)	32/229 (13.97%)	
HIT‐6, mean (SD)	66.79 (7.314)	66.90 (6.96)	0.882
No impact, *n*/*N* (%)	5/167 (2.99%)	3/202 (1.49%)	0.322
Mild, *n*/*N* (%)	3/167 (1.80%)	1/202 (0.50%)	0.230
Moderate, *n*/*N* (%)	5/167 (2.99%)	10/202 (4.95%)	0.343
Severe, *n*/*N* (%)	152/165 (92.12%)	185/199 (92.96%)	0.760
Comorbidity, *n*/*N* (%)			
Anxiety*n*/*N* (%)	94/179 (52.51%)	131/228 (57.46%)	0.320
Depression *n*/*N* (%)	80/179 (44.69%)	107/229 (4672%)	0.683
Insomnia *n*/*N* (%)	92/178 (51.69%)	98/229 (42.74%)	0.075
HBP *n*/*N* (%)	18/178 (10.11%)	48/228 (21.05%)	0.003*
Obesity *n*/*N* (%)	28/175 (16%)	52/227 (22.91%)	0.085
Migraine type, *n*/*N* (%)			
CM, *n*/*N* (%)	122/179 (68.17%)	188/229 (82.10%)	0.001*
HFEM,*n*/*N* (%)	57/179 (31.84%)	41/229 (17.90%)	
Aura, *n*/*N* (%)	54/179 (30.17%)	71/229 (31%)	0.856
MO, *n*/*N* (%)	102/178 (57.30%)	168/229 (73.36%)	0.002*
Migraine duration, years, mean (SD)	29.28 (13.09)	29.70 (12.76)	0.75
Time of CM, months, mean (SD)	119.68 (88.646)	123.19 (95.953)	0.752
MHD, mean (SD)	21.14 (7.835)	23.13 (7.270)	0.009*
MMD, mean (SD)	13.77 (6.078)	15.97 (7.516)	0.002*
Prior number of prophylactic treatments, mean (SD)	8.51 (3.739)	8.73 (3.710)	0.557
Number of concomitant prophylactic treatments at onset, mean (SD)	1.79 (1.026)	1.45 (0.785)	<0.001*
Anti‐CGRP treatment duration, mean (SD)	16.45 (8.298)	15.77 (8.786)	0.423
Reduction in MMD, mean (SD)	8.99 (6.667)	9.04 (7.467)	0.953
MO post anti‐CGRP, *n*/*N* (%)	22/179 (12.29%)	72/226 (31.86%)	<0.001*
MHD post anti‐CGRP, mean (SD)	9.62 (8.56)	14.13 (10.21)	<0.001*
MMD days post anti‐CGRP, mean (SD)	4.99 (3.96)	8.26 (7.67)	<0.001*
Number of concomitant prophylactic treatments after anti‐CGRP, mean (SD)	0.86 (1.03)	1.59 (0.91)	<0.001*

Abbreviations: CGRP, calcitonin gene‐related peptide; CM, chronic migraine; HBP, high blood pressure; HFEM, high‐frequency episodic migraine; HIT‐6, Headache Impact Test; MHD, monthly headache days; MMD, monthly migraine days.

*p < 0.001.

Of the 408 patients enrolled in the study, 179 individuals (43.87%) were able to withdraw their preventive treatment. Among these, 85 patients (20.83%) achieved complete withdrawal, whereas 94 patients (23.04%) experienced partial withdrawal.

The specific preventive treatments with the highest rates of total withdrawal were valproic acid (66.67%), flunarizine (50%), and lamotrigine (40%). In terms of overall withdrawals, the most notable contributors were OnabotA, accounting for 17.54% of total withdrawals; beta‐blockers at 14.62%; and amitriptyline at 12.28%.

**FIGURE 2:**
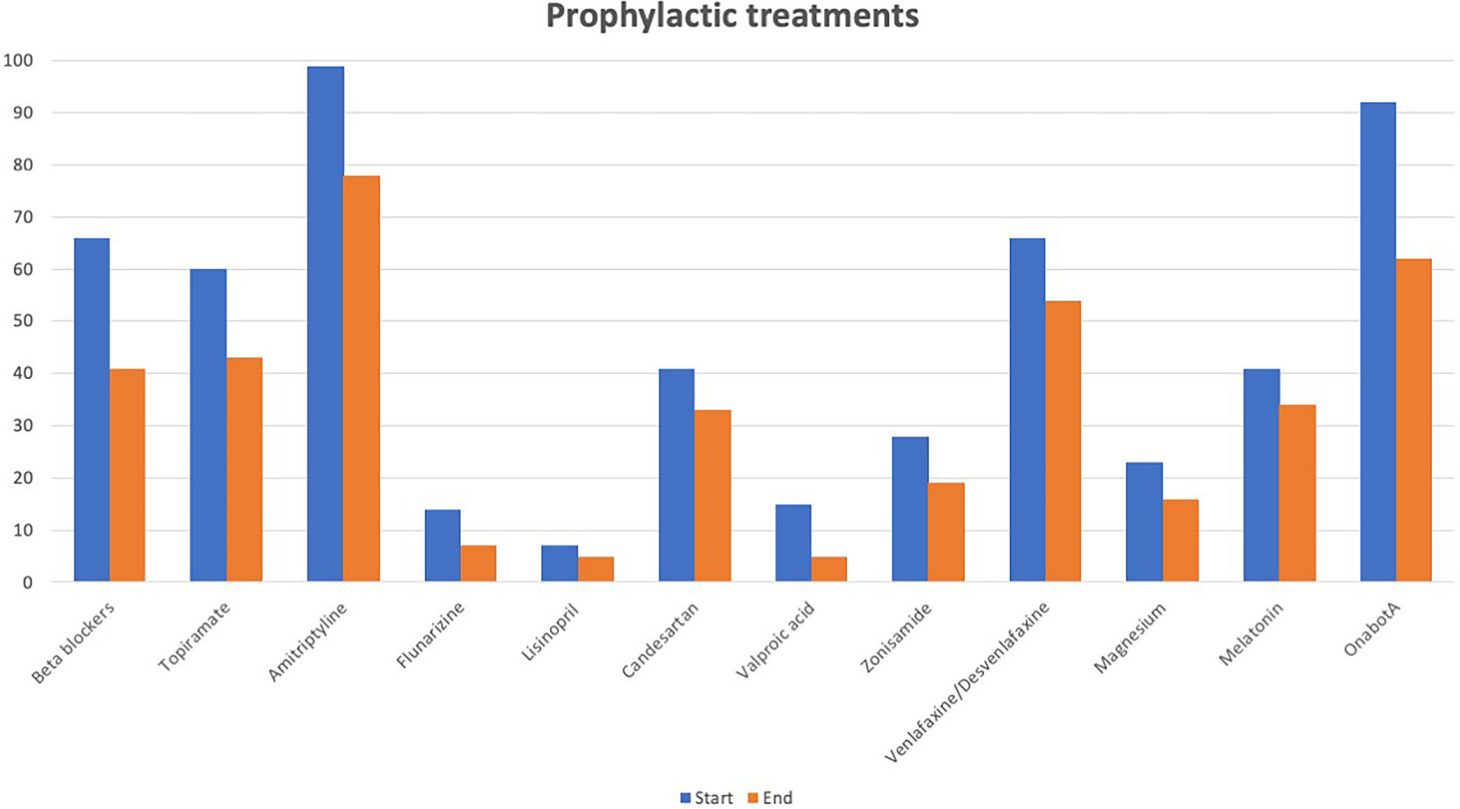
Number of patients with each preventive treatment, at the beginning of anti‐calcitonin gene‐related peptide antibodies and at the end (inclusion in this study). OnabotA, onabotulinumtoxinA.

**FIGURE 3:**
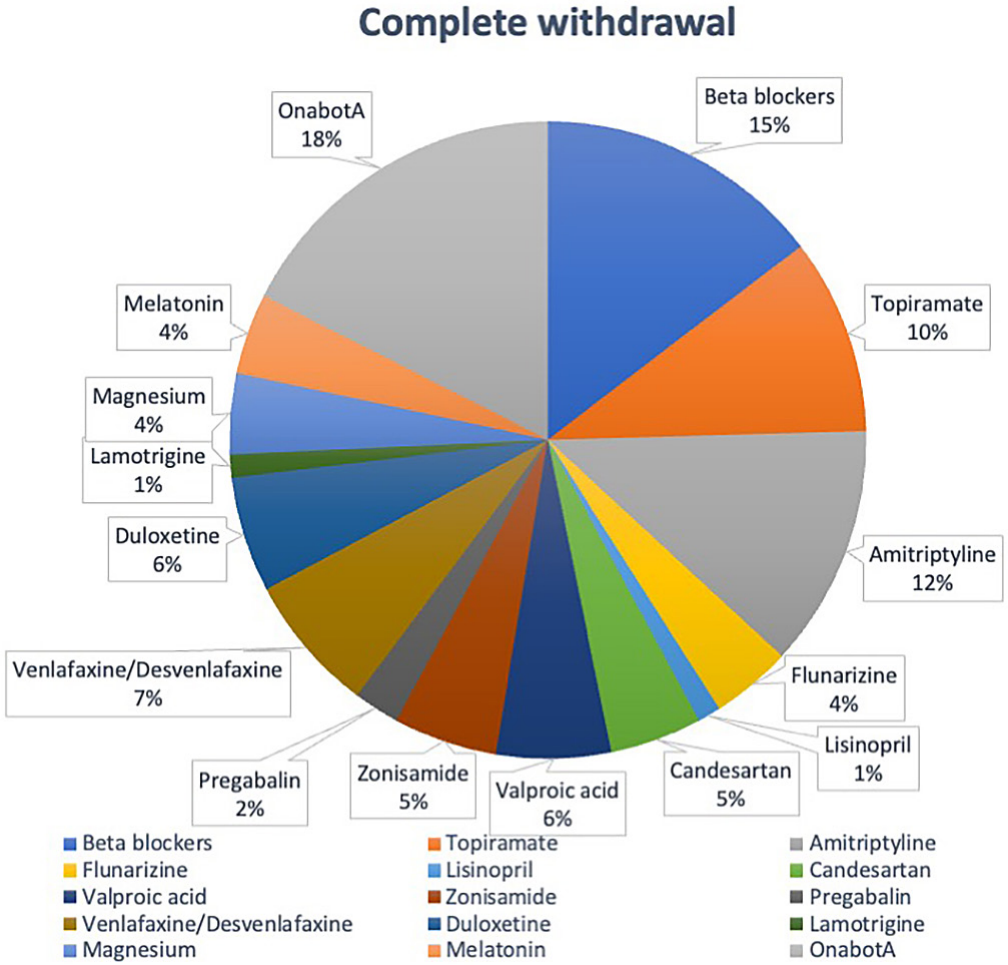
Complete withdrawal percentage over total concomitant treatments. OnabotA, onabotulinumtoxinA.

Additional comprehensive information regarding the total withdrawal rates for each preventive treatment can be found in Table 2, along with visual representations in Figures 2 and 3 and Table S1.

**TABLE 2:** Multivariate analysis of variables associated with withdrawal.

Baseline characteristics	Exp (*B*)	95% CI		*p*
		Inferior	Superior	
Age	0.984	0.963	1.006	0.148
Insomnia	1.316	0.818	2.118	0.258
HBP	0.396	0.184	0.854	0.018*
MHD at baseline	1.001	0.958	1.046	0.975
MMD at baseline	0.998	0.946	1.053	0.488
Prior number of prophylactics	1.830	1.384	2.419	<0.0001**
Obesity	0.755	0.411	1.388	0.366
Migraine type	1.403	0.686	2.869	0.354
MO	0.690	0.403	1.182	0.176
MHD post‐anti‐CGRP	0.988	0.955	1.022	0.478
MMD post‐anti‐CGRP	0.913	0.862	0.967	0.002*
MO post‐anti‐CGRP	0.609	0.282	1.314	0.206
Intercept	2.161			0.324

Abbreviations: CGRP, calcitonin gene‐related peptide; CI, confidence interval; HBP, high blood pressure; MHD, monthly headache days; MMD, monthly migraine days; MO, medication overuse.

*p < 0.05; **p < 0.001.

**FIGURE 4:**
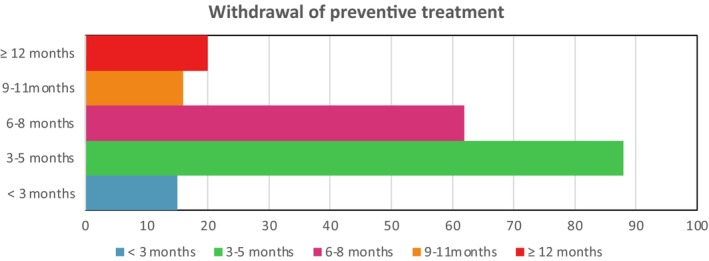
Withdrawal (partial or total) of preventive treatment in patients under anti‐calcitonin gene‐related peptide therapies. OnabotA, onabotulinumtoxinA.

Preventive treatments were most frequently withdrawn 3–5 months after starting treatment with anti‐CGRP antibodies. In this time interval, 88 of them (43.78%) were withdrawn totally or partially, followed by the period of 6–8 months, with 62 completed treatments (30.85%). These results are shown in Figure 4.

In 112 of 408 (27.45%), it was maintained exclusively due to comorbidity and in 117 of 408 (28.6%) due to partial effectiveness. Moreover, the percentage of withdrawal was higher in responders (50% response rate considering MMD) compared to nonresponders (61/253 [24%] vs. 21/145 [14%], *p* < 0.02).

The baseline characteristics associated with nonwithdrawal were insomnia, hypertension and obesity, chronic migraine, and MO (Table 1). In the multivariate analysis, the absence of high blood pressure, greater number of preventive treatments at anti‐CGRP onset, and lower number of migraine days/month after anti‐CGRP treatment were independently associated with withdrawal of the treatment (*p* < 0.05).

## DISCUSSION

To the best of our understanding, this marks the inaugural multicenter study investigating the influence of anti‐CGRP treatment on the administration of concurrent therapies within a real‐world context. Notably, our observations revealed that >40% of patients—split almost evenly between partial and complete withdrawals—managed to discontinue at least one preventive treatment upon being treated with anti‐CGRP therapies.

In clinical practice, attempts should be made to limit the exposure time to preventive drugs due to the prevalence of adverse effects [24]. This is particularly significant for older patients, who typically require multiple concomitant medications. It is worth noting that the novel anti‐CGRP drugs have demonstrated safety in the elderly population [25]. The ability to discontinue preventive medication gains heightened importance when considering the prolonged duration of migraines, which averages approximately 30 years in our study. These patients often undergo trials of numerous preventive treatments (almost nine on average), yet find none to be entirely effective. Consequently, >90% of them continue to experience severe migraine, with a high impact on their quality of life, as indicated by the HIT‐6 scale score. Despite the treatment‐resistant nature of some patients, anti‐CGRP therapies continue to exhibit effectiveness, not only among individuals with refractory migraines but also in those with migraine and MO [12, 26, 27, 28].

Our study's findings align with previous research on botulinum toxin type A (BTX‐A) and concurrent oral treatments in CM, where nearly half of BTX‐A‐treated patients were able to discontinue at least one oral medication [15]. Anti‐CGRP therapies demonstrate prompt efficacy in terms of reducing MMD and MHD from the first month of treatment [12, 29]. Notably, the withdrawal of concurrent treatments was predominantly observed after 3 months. This observation might be attributed to the early effectiveness of anti‐CGRP therapies, extending up to 6 months. According to European guidelines, if there is a 30% improvement within the first 3 months, there is a potential for further improvement in the subsequent trimester [14]. These results can be related with effectiveness that is observed in the beginning of anti‐CGRP therapies and up to 6 months. Hence, the decline in concurrent preventive treatments could also serve as an indirect indicator of the continued efficacy of the recently introduced anti‐CGRP therapies.

Furthermore, our investigation highlighted that BTX‐A, beta‐blockers, and amitriptyline were the treatments most frequently withdrawn. This trend can be attributed to their widespread utilization among patients. Regarding BTX‐A, its withdrawal could be connected to its specific use, as it is not commonly employed to manage comorbidities like high blood pressure or anxiety, which might otherwise warrant concurrent use in different scenarios. Although our study did not identify a statistically significant association between these treatments and anxiety or depression, it is worth noting the potential for a bidirectional relationship that merits further exploration [30].

Additionally, a higher count of preventive treatments initiated alongside anti‐CGRP therapy was independently connected with the discontinuation of treatment (*p* < 0.05). This finding further supports the notion of a relationship between the withdrawal of concurrent treatments and the effectiveness of anti‐CGRP therapy.

We also observed that the absence of high blood pressure was linked to the withdrawal of treatments. This association could potentially be attributed to the circumstance that most concurrent treatment use arises in the context of managing comorbidities, rather than as a response to ineffectiveness [7]. Moreover, a greater number of preventive treatments at anti‐CGRP onset might be related to the selection of patients who are refractory to other preventive pathways. Finally, lower number of MMD after anti‐CGRP treatment was independently associated with withdrawal of the treatment (*p* < 0.05), which supports the association between concomitant treatment withdrawal and anti‐CGRP effectiveness.

Regarding the main limitations of the study, the study design does not allow evaluation of intermediate time points nor longer periods regarding the lack of follow‐up; moreover, there is no robust evidence to guide the cessation of preventive treatment, so there might be differences in criteria between centers and between specialists, although all the patients were being attended at headache units, which might minimize the variability. In addition, we cannot predict the need to reintroduce the preventive treatment in the long term, although most of the patients did not need to reintroduce treatment after 3 months of follow‐up. Additionally, although our sample resembles the population of patients receiving anti‐CGRP monoclonal antibodies in Spain [12], the characteristics of patients without concomitant therapies at anti‐CGRP onset were not evaluated. Therefore, further studies are necessary on the pathophysiological mechanisms that will accompany the end of preventive treatments or the identification of prognostic biomarkers that can predict the evolution of the disease after the discontinuation of preventive treatment.

## CONCLUSIONS

As a whole, these results show that anti‐CGRP antibodies allow the withdrawal of associated preventive treatment in a significant percentage of patients, which supports their effectiveness in real‐life conditions and prior evidence of the importance of concomitant medication withdrawal in effective measurement of novel therapies, which should be taken into account together with patients' reported outcomes to identify other effectiveness measurements and to reduce adverse events.

## AUTHOR CONTRIBUTIONS

**Alicia Gonzalez‐Martinez:** Conceptualization; funding acquisition; investigation; writing – original draft; methodology; validation; visualization; writing – review and editing; software; formal analysis; project administration; data curation; supervision; resources. **Ana Beatriz Gago‐Veiga:** Conceptualization; writing – original draft; methodology; writing – review and editing; formal analysis; supervision; data curation; resources; investigation; funding acquisition; validation; visualization; software; project administration. **Noelia Lopez‐Alcaide:** Investigation; writing – review and editing; data curation. **Sonia Quintas:** Writing – review and editing; investigation; methodology; formal analysis; data curation; resources; supervision; software; visualization. **Iris Fernández Lázaro:** Writing – review and editing; investigation; data curation; formal analysis; project administration; supervision. **Javier Casas‐Limón:** Writing – review and editing; investigation; data curation. **Carlos Calle:** Writing – review and editing; investigation; data curation. **Germán Latorre:** Writing – review and editing; investigation; data curation. **Nuria González‐García:** Investigation; writing – review and editing; data curation. **Jesús Porta‐Etessam:** Writing – review and editing; investigation; data curation. **Jaime Rodriguez‐Vico:** Writing – review and editing; investigation; data curation. **Alex Jaimes:** Writing – review and editing; investigation; data curation. **Andrea Gómez García:** Investigation; writing – review and editing; data curation. **David García‐Azorín:** Investigation; writing – review and editing; data curation. **Ángel Luis Guerrero‐Peral:** Investigation; writing – review and editing; data curation. **Álvaro Sierra:** Investigation; writing – review and editing; data curation. **Alberto Lozano Ros:** Investigation; writing – review and editing; data curation. **Antonio Sánchez‐Soblechero:** Writing – review and editing; investigation; data curation. **Javier Díaz‐de‐Teran:** Writing – review and editing; investigation; data curation. **Javier Membrilla:** Funding acquisition; data curation; writing – review and editing. **Cristina Treviño:** Data curation; investigation; writing – review and editing.

## CONFLICT OF INTEREST STATEMENT

A.G.‐M. has received speaker honoraria from Teva. D.G.‐A. reports payment or honoraria for lectures from Teva, Lilly, Allergan‐Abbvie, Novartis, and Lundbeck, and has served on an advisory board for Allergan‐Abbvie. J.D.‐d.‐T. has received speaker honoraria from Novartis, Lilly, and Teva. S.Q. has received speaker honoraria from Lilly and Novartis. J.C.‐L. has received speaker honoraria from Novartis, Eli Lilly, and Teva. G.L. has received speaker honoraria from Lilly, Novartis, Teva, Chiesi, and Allergan. Á.L.G.‐P. has received has received honoraria from Lilly, Teva, Novartis, Allergan‐Abbvie, and Exeltis, and research support from Allegan‐Abbvie and Teva. C.T. has received speaker honoraria from Teva. A.B.G.‐V. has received honoraria from Lilly, Novartis, Teva, Abbvie‐Allergan, Exeltis, and Chiesi. A.L.R. has received speaker honoraria from Teva. A.S.‐S. has received speaker honoraria from Teva. None of the other authors has any conflict of interest to disclose.

## Supporting information


TABLE S1

## Data Availability

The data that support the findings of this study are available from the corresponding author upon reasonable request.
