# Supplementary material for: Evaluation of the concomitant use of prophylactic treatments in patients with migraine under anti‐calcitonin gene‐related peptide therapies: The PREVENAC study
Source: Eur J Neurol. 2024 Feb 7;31(5):e16215. doi: 10.1111/ene.16215 (PMC11235635; doi:10.1111/ene.16215)
Supplement: Supplementary file 1 — TABLE S1 [file ENE-31-e16215-s001.docx]

**Table 1S. Comparison of the percentage of patients under the most common preventive treatment, at the beginning of anti-CGRP monoclonal antibodies treatment and at the end (time of inclusion in this study).**

| **Prophylactic treatment** | **Start** | **End** | **P-Value** |
| --- | --- | --- | --- |
| **Beta blockers** | **66 (16%)** | **41 (10%)** | **<0.05*** |
| **Topiramate** | **60 (15%)** | **43 (11%)** | **<0.05*** |
| **Amitriptyline** | **99 (24%)** | **78 (19%)** | **<0.05*** |
| **OnabotA** | **92 (22%)** | **62 (15%)** | **<0.05*** |
| **Venlafaxine/Desvenlafaxine** | **66 (16%)** | **54 (13%)** | **<0.05*** |
| **Candesartan** | **41 (10%)** | **33 (8%)** | **<0.05*** |
| Melatonin | 41 (10%) | 34 (8%) | 0.057 |
| **Zonisamide** | **28 (7%)** | **19 (5%)** | **<0.05*** |
| **Magnesium** | **23 (5%)** | **16 (4%)** | **<0.05*** |
| **Valproic acid** | **15 (4%)** | **5 (2%)** | **<0.05*** |
| **Flunarizine** | **14 (3%)** | **7 (2%)** | **<0.05*** |
| Lisinopril | 7 (2%) | 5 (1%) | 0.157 |

McNemar test for paired groups was performed to compare the percentage of each prophylactic drug before and after CGRP treatment. *P<0.05.
